# Long-Term Urban Particulate Air Pollution, Traffic Noise, and Arterial Blood Pressure

**DOI:** 10.1289/ehp.1103564

**Published:** 2011-08-09

**Authors:** Kateryna Fuks, Susanne Moebus, Sabine Hertel, Anja Viehmann, Michael Nonnemacher, Nico Dragano, Stefan Möhlenkamp, Hermann Jakobs, Christoph Kessler, Raimund Erbel, Barbara Hoffmann, on behalf of the Heinz Nixdorf Recall Study Investigative Group

**Affiliations:** 1 Leibniz Research Institute for Environmental Medicine, Düsseldorf, Germany; 2 Institute for Medical Informatics, Biometry, and Epidemiology, and; 3 West German Heart Centre of Essen, University Hospital Essen, University of Duisburg-Essen, Essen, Germany; 4 Rhenish Institute for Environmental Research, University of Cologne, Cologne, Germany; 5 Medical School, Heinrich Heine University of Düsseldorf, Düsseldorf, Germany

**Keywords:** atherosclerosis, environmental epidemiology, hypertension, particulate matter, traffic emissions

## Abstract

Background: Recent studies have shown an association of short-term exposure to fine particulate matter (PM) with transient increases in blood pressure (BP), but it is unclear whether long-term exposure has an effect on arterial BP and hypertension.

Objectives: We investigated the cross-sectional association of residential long-term PM exposure with arterial BP and hypertension, taking short-term variations of PM and long-term road traffic noise exposure into account.

Methods: We used baseline data (2000–2003) on 4,291 participants, 45–75 years of age, from the Heinz Nixdorf Recall Study, a population-based prospective cohort in Germany. Urban background exposure to PM with aerodynamic diameter ≤ 2.5 μm (PM_2.5_) and ≤ 10 μm (PM_10_) was assessed with a dispersion and chemistry transport model. We used generalized additive models, adjusting for short-term PM, meteorology, traffic proximity, and individual risk factors.

Results: An interquartile increase in PM_2.5_ (2.4 μg/m^3^) was associated with estimated increases in mean systolic and diastolic BP of 1.4 mmHg [95% confidence interval (CI): 0.5, 2.3] and 0.9 mmHg (95% CI: 0.4, 1.4), respectively. The observed relationship was independent of long-term exposure to road traffic noise and robust to the inclusion of many potential confounders. Residential proximity to high traffic and traffic noise exposure showed a tendency toward higher BP and an elevated prevalence of hypertension.

Conclusions: We found an association of long-term exposure to PM with increased arterial BP in a population-based sample. This finding supports our hypothesis that long-term PM exposure may promote atherosclerosis, with air-pollution–induced increases in BP being one possible biological pathway.

High blood pressure (BP) and hypertension have been identified as the leading risk factors for cardiovascular mortality and, together, as the third most important cause of disability in the developed world (Ezzati et al. 2002). Chronically elevated BP is an important determinant for the development and progression of atherosclerosis, the underlying pathology for most cardiovascular events. Recent studies suggest that long-term exposure to particulate matter (PM) contributes to atherogenesis and that the induction of a chronically elevated BP could be one biological pathway by which PM exerts its influence on atherosclerosis (Adar et al. 2010; Allen et al. 2009; Bauer et al. 2010; Diez Roux et al. 2008; Hoffmann et al. 2007; Künzli et al. 2010).

Increasing evidence suggests that short-term increases in PM air pollution lead to acute but transient increases in arterial BP (Brook et al. 2011; Chuang et al. 2010; Delfino et al. 2010; Dvonch et al. 2009; Mordukhovich et al. 2009; Zanobetti et al. 2004). Less is known regarding whether long-term exposure to air pollution can chronically raise BP and induce hypertension. Recent epidemiologic studies provide preliminary evidence showing positive associations between medium- and long-term residential exposures to fine PM and BP and left ventricular mass (Auchincloss et al. 2008; Chuang et al. 2011; Van Hee et al. 2009).

Although biological mechanisms for the effects of PM on BP have not yet been established, several pathways have been suggested, including local and systemic inflammation and oxidative stress, autonomic imbalance, and endothelial dysfunction (Brook 2007; Brook et al. 2009; Mills et al. 2009).

The composition of urban PM air pollution is determined by multiple sources, including regionally transported PM, local industrial sources, home heating, and traffic. Traffic is also an important source of long-term residential noise exposure, which has been suggested to increase arterial BP and promote hypertension (Babisch 2006). When studying the effects of long-term urban air pollution on BP, it is therefore necessary to account for the effects of road traffic noise when estimating the effects of air pollution. One possible solution is to measure urban background PM concentration and small-scale indicators of traffic exposure and traffic noise separately. This separation can be achieved because *a*) traffic-related PM is only one component among many that determine urban background concentration of PM and *b*) the dispersions of traffic-related PM and traffic noise follow different patterns.

In our study, we investigated the cross-sectional association of long-term urban background PM with arterial BP and hypertension in a population-based sample, taking long-term traffic noise and short-term variations of air pollution into account. We used data from a large, well-characterized population-based cohort, residing in a densely populated area in West Germany. The availability of different exposure indicators, including a state-of-the-art dispersion and chemistry transport model for the assessment of urban background PM exposure, in addition to small-scale indicators of traffic and traffic noise exposures, enabled us to account for the effects of road traffic noise when estimating the effects of PM.

## Materials and Methods

*Population.* We used baseline data (2000–2003) from the Heinz Nixdorf Recall Study, a population-based, prospective cohort study in the highly urbanized Ruhr area in western Germany (Schmermund et al. 2002). In total, 4,814 men and women 45–75 years of age participated at baseline. The study was approved by the local institutional review board. All study participants gave informed consent.

*Environmental data.* We applied a validated dispersion and chemistry transport model [the European Air Pollution Dispersion (EURAD) model system] to model daily mass concentrations of PM with an aerodynamic diameter ≤ 2.5 μm (PM_2.5_) and ≤ 10 μm (PM_10_) on a grid of 1 km^2^ (Ebel et al. 2007; Memmesheimer et al. 2004). Official emission inventory data on a scale of 1 km^2^, hourly meteorology, and regional topography were used in the model. The primary model output was then calibrated with measured PM_10_ concentrations from routine monitoring stations. The daily surface concentrations of PM and ozone (O_3_) for the period from November 2000 through July 2003 were assigned to the participants’ addresses using a geographic information system (ArcView, version 9.2; ESRI, Redlands, CA, USA). We calculated the mean of the 365 daily PM values before the baseline examination date for each participant. For participants examined before November 2001 [*n* = 1,162 of 4,814 (24.1%)], we used the mean of 365 daily values starting from the first available PM measurement.

*Traffic proximity.* We assessed residential proximity to the nearest major road using digitalized maps (ArcView) and daily traffic counts for 2000 [obtained by request from North Rhine-Westphalia State Agency for Nature, Environment and Consumer Protection (LANUV), Recklinghausen, Germany]. A major road was defined as a road section in the upper quartile of the daily traffic count in the study area (> 22,980 vehicles/day for general traffic, > 756 vehicles/day for heavy-duty traffic). We categorized proximity to the road as ≤ 50 m, 51–100 m, 101–200 m, and > 200 m (reference category).

*Road traffic noise.* Long-term road noise was modeled according to the Directive 2002/49/EC of the European Parliament and of the Council (European Commission 2002) as a weighted day-evening-night 24-hr average sound level (*L*_den_). The following parameters were considered for noise level modeling: small-scale topography of the area, dimensions of buildings, noise barriers, street axis, vehicle-type–specific traffic density, speed limit, and type of street surface. The following formula was used:


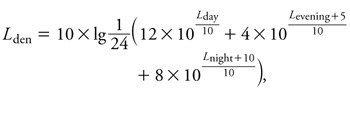


where *L*_day_, *L*_evening_, and *L*_night_ are the A-weighted long-term average sound level (in decibels) determined over all the day, evening, and night periods of a year, respectively. Exposure to long-term road traffic noise was categorized as ≤ 55 dB (reference), > 55 to ≤ 60 dB, > 60 to ≤ 65 dB, and > 65 dB.

*Outcome.* BP was measured with an automated oscillometric device (model HEM-705CP; OMRON Corp., Hoofddorp, the Netherlands), using standardized recording protocols as described previously (Stang et al. 2006). For the analyses, we calculated the mean of the second and third of three measurements [see Supplemental Material (http://dx.doi.org/10.1289/ehp.1103564)]. Medication use during last 7 days was categorized according to the Anatomical Therapeutic Chemical Classification System of the World Health Organization Collaborating Centre for Drug Statistics Methodology (2006). We defined antihypertensive medication as current intake of the following classes of drugs or any combination of them: diuretics, ®-blockers, angiotensin-converting enzyme inhibitors, angiotensin-receptor antagonists, calcium-channel blockers, 〈-blockers, centrally active antihypertensive drugs, and hydralazine. We defined hypertension as systolic BP ≥ 140 mmHg or diastolic BP ≥ 90 mmHg, or current use of antihypertensive therapy (Chobanian et al. 2003).

*Other covariates.* We assessed current and previous smoking, the lifetime cumulative exposure in pack-years, and passive smoking exposure. Amount of alcohol intake was given as the number of drinks per week (one drink defined as 0.25 L beer, 0.1 L wine, or 0.02 L spirits), categorized as 0, 1–3, > 3 drinks per week. Participants’ physical activity was expressed as weekly energy expenditure in metabolic equivalents. We defined diabetes mellitus as prior physician diagnosis of diabetes mellitus, use of antidiabetic drugs, random blood glucose ≥ 11.1 mmol/L, or fasting blood glucose ≥ 7 mmol/L. Individual-level socioeconomic status (SES) was assessed as years of formal education (United Nations Educational, Scientific, and Cultural Organization 1997), and economic activity (i.e., employed, retired, unemployed, or economically inactive).

*Statistical methods.* We included participants with nonmissing information on exposure, outcome, and covariates. Correlations between PM exposures, traffic proximity, road traffic noise, and short-term environmental variables were assessed with Spearman’s rank correlation coefficient. We assessed the association of PM with BP and hypertension using generalized additive models and logistic models, respectively. PM_2.5_ and PM_10_ were included as linear terms, based on prior evidence of a linear exposure–response relationship between PM and cardiovascular outcomes. We identified possible confounders using causal graphs (Glymour and Greenland 2008). We specified the most likely temporal relations between variables based on prior biological and epidemiologic knowledge and derived adjustment sets [for additional information on model specifications, see Supplemental Material, Figure 1 and pp. 6–7 (http://dx.doi.org/10.1289/ehp.1103564)]. Specifically, we included the main exposure (1-year mean PM_2.5_ or PM_10_), short-term environmental exposures (spatially resolved 3-day lag of PM, 3-day mean of temperature, season, and time trend), traffic proximity, and area-level variables (city, area) in the basic model. The main model additionally included sex, age, and individual-level covariates [body mass index (BMI), alcohol consumption, smoking, passive smoking, and physical activity] instead of area-level variables. In extended models we also included traffic noise and individual SES, antihypertensive medication, diabetes mellitus, and short-term O_3_ exposure (4-day mean). To study the between- and within-area effects, we conducted an extended analysis with a model that included both area-level and individual-level confounders, thereby reflecting the within-city effect of the basic model in a fully adjusted model. To investigate the effect of long-term PM exposure in participants without antihypertensive medication, we conducted a stratified analysis according to intake of antihypertensive medication. Continuous covariates that did not display departure from a linear relationship with the outcome were entered as linear terms. Time trend, age, BMI, and pack-years of smoking (in the analyses of diastolic BP) were handled as penalized cubic splines (Hastie and Tibshirani 2007).

**Figure 1:**
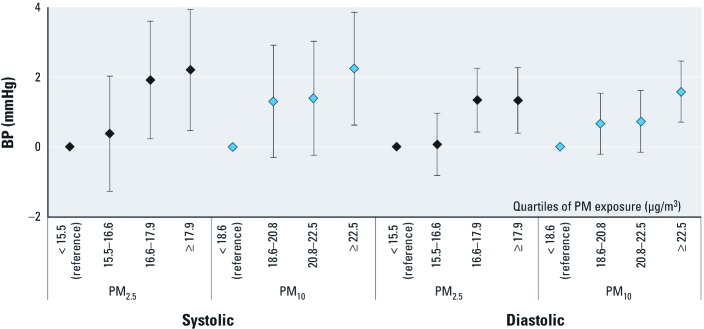
Estimated absolute increase in arterial BP (mmHg) (with 95% CI) by quartiles of yearly mean PM exposure (μg/m^3^) in participants of the Heinz Nixdorf Recall Study (*n* = 4,291); reference is quartile 1; adjusted for short-term PM, temperature, season, time trend, traffic proximity, sex, age, BMI, alcohol consumption, smoking, passive smoking, and physical activity.

For the exposure–response analyses, PM was divided into quartiles, with the lowest as the reference category. We explored possible exposure effect modification by covariates using product terms. For this, continuous variables were dichotomized at the median.

In further sensitivity analyses, we estimated the PM effect after excluding 500 participants exposed to high noise levels (*L*_den_ > 65 dB) from the analysis. Statistical analyses were performed using SAS software (version 9.2; SAS Institute Inc., Cary, NC, USA). All effects are given per interquartile range (IQR) of the exposure if not specified otherwise.

## Results

We included 4,291 participants with nonmissing values on exposure, outcome, and covariates. The 523 excluded participants did not differ substantially from those included, although they had slightly higher rates of alcohol consumption and higher prevalence of current smoking and environmental tobacco exposure [see Supplemental Material, Table 1 (http://dx.doi.org/10.1289/ehp.1103564)]. Mean systolic and diastolic BP levels of the study participants were 132.7 and 81.2 mmHg, respectively (Table 1). Hypertension (57.0%) and use of antihypertensive medication (36.1%) were highly prevalent in this elderly population. Mean 1-year PM_2.5_ and PM_10_ exposure for the study population were 16.7 and 20.7 μg/m^3^, respectively. About 16% of study participants resided within 200 m of a highly trafficked road. One-year mean values of PM_2.5_ and PM_10_ were highly correlated with each other (Spearman correlation coefficient = 0.84; see Supplemental Material, Table 2). Correlations of long-term PM exposure with individual daily PM values were low (0.15–0.26), as were correlations of PM with proximity to high traffic and road traffic noise (0.07–0.24).

**Table 1:** Baseline characteristics (2000–2003) of the analyzed (*n* = 4,291) participants of the Heinz Nixdorf Recall Study.

Variable	Mean ± SD (IQR) or *n* (%)
Long-term exposure	
Mean PM_2.5_ (μg/m^3^)	16.7 ± 1.6 (2.4)
Mean PM_10_ (μg/m^3^)	20.7 ± 2.6 (3.9)
Traffic proximity, general (m)	
≤ 50	131 (3.1)
> 50–100	178 (4.1)
> 100–200	365 (8.5)
> 200	3,617 (84.3)
24-hr mean noise [*L*_den_, dB(A)]	
≤ 55	2,757 (64.3)
> 55–60	542 (12.6)
> 60–65	490 (11.4)
> 65	502 (11.7)
Short-term environmental variables	
PM_2.5_ (3-day lag, μg/m^3^)	16.5 ± 9.5
PM_10_ (3-day lag, μg/m^3^)	20.5 ± 11.3
Temperature (3-day mean, °C)	10.6 ± 7.4
O_3_ (4-day mean, μg/m^3^)	37.4 ± 20.0
Outcome	
Systolic BP (mmHg)	132.7 ± 20.6
Diastolic BP (mmHg)	81.2 ± 10.8
Hypertension	2,444 (57.0)
Antihypertensive medication	1,550 (36.1)
Covariates	
Male sex	2,147 (50.0)
Age (years)	59.7 ±7.8
Coronary heart disease	290 (6.8)
Diabetes mellitus	594 (13.8)
BMI (kg/m^2^)	27.9 ± 4.6
Weekly physical activity (10^3^ metabolic equivalents)	1.3 ± 2.1
Weekly alcohol intake (drinks)	
0	2,131 (49.7)
1–3	749 (17.5)
> 3	1,411 (32.9)
Smoking	
Never	1,789 (41.7)
Ex-smoker	1,513 (35.3)
Current smoker	989 (23.0)
Education	
≤ 10 years	461 (10.7)
11–13 years	2,390 (55.7)
14–17 years	970 (22.6)
≥ 18 years	470 (11.0)
Economic activity	
Employed	1,716 (40.0)
Inactive/homemaker	590 (13.8)
Retired	1,719 (40.1)
Unemployed	266 (6.2)

**Table 2:** Estimated absolute increase in arterial BP (mmHg) (with 95% CI) per corresponding IQR of long-term exposure to PM_2.5_ and PM_10_ (µg/m^3^) (*n* = 4,291).

Change in arterial BP (mmHg)						
Per IQR of PM_2.5_ (2.4 μg/m^3^)			Per IQR of PM_10_ (3.9 μg/m^3^)		
Model	Systolic BP	Diastolic BP	Systolic BP	Diastolic BP
Crude		1.0 (0.1, 2.0)		0.3 (–0.2, 0.8)		1.3 (0.3, 2.2)		0.5 (0.0, 1.0)
Basic*a*		1.3 (–0.4, 3.0)		0.7 (–0.2, 1.6)		0.3 (–1.3, 1.9)		0.3 (–0.5, 1.2)
+ Noise*b* and SES		1.5 (–0.1, 3.2)		0.7 (–0.2, 1.6)		0.6 (–0.9, 2.1)		0.3 (–0.5, 1.1)
Main*c*		1.4 (0.5, 2.3)		0.9 (0.4, 1.4)		1.1 (0.2, 2.0)		0.8 (0.3, 1.2)
+ Noise*b* and SES		1.4 (0.5, 2.4)		0.8 (0.3, 1.4)		1.1 (0.2, 2.0)		0.7 (0.3, 1.2)
Extended = main								
+ Medication		1.4 (0.5, 2.3)		0.9 (0.4, 1.4)		1.1 (0.2, 2.0)		0.8 (0.3, 1.2)
+ Diabetes		1.4 (0.5, 2.4)		0.9 (0.4, 1.4)		1.1 (0.2, 2.0)		0.8 (0.3, 1.2)
+ O_3_*d*		1.3 (0.3, 2.2)		0.8 (0.3, 1.3)		1.0 (0.1, 1.9)		0.7 (0.2, 1.2)
+ City, area		1.5 (–0.1, 3.1)		0.7 (–0.2, 1.5)		0.6 (–0.8, 2.1)		0.5 (–0.3, 1.3)
**a**Adjusted for short-term PM, temperature, season, time trend, traffic proximity, city, and area of residence. **b**Models adjusting for traffic noise do not include traffic proximity. **c**Adjusted for short-term PM, temperature, season, time trend, traffic proximity, sex, age, BMI, alcohol consumption, smoking, passive smoking, and physical activity. **d**Because of high correlation with O_3_, short-term temperature was excluded from this adjustment set.								

We found an association of long-term PM exposure with arterial BP, which was consistent across a wide range of different model specifications (Table 2) [for the full results of the main model for systolic BP, see Supplemental Material, Table 3 (http://dx.doi.org/10.1289/ehp.1103564)]. In the crude model, systolic BP increased by 1.0 mmHg [95% confidence interval (CI): 0.1, 2.0 mmHg], and diastolic BP by 0.3 mmHg (95% CI: –0.2, 0.8 mmHg) per 2.4-μg/m^3^ increase in 1-year mean PM_2.5_. Adjustment for short-term exposures and area-level variables led to small increases in the effect estimates. After adjusting for short-term exposures, traffic proximity, and individual-level covariates (main model), estimated effects on systolic and diastolic BP were 1.4 mmHg (95% CI: 0.5, 2.3 mmHg) and 0.9 mmHg (95% CI: 0.4, 1.4 mmHg), respectively. Temperature, female sex, and current smoking were inversely associated with systolic BP, whereas age, alcohol (> 3 drinks/week), BMI, and time trend showed positive association (see Supplemental Material, Table 3). Using road traffic noise instead of traffic proximity and including individual SES in the main model did not change the estimates noticeably (Table 2). When we used traffic counts within 100 m of the residence instead of traffic proximity, estimates remained unchanged (data not shown). Adjusting for antihypertensive medication, diabetes, short-term O_3_, and area-level variables did not alter the estimated effect of long-term PM_2.5_. Adjusting for the same variables also had little impact on estimated effects for PM_10_, with the exception of area-level variables, which attenuated estimated effects for PM_10_: 1.1 (95% CI: 0.2, 2.0) mmHg change in systolic BP in the main model and 0.6 (95% CI: –0.8, 2.1) mmHg when additionally adjusted for city and area.

When restricting the study population to participants who did not use antihypertensive medication (*n* = 2,741), estimates for long-term PM_2.5_ were marginally lower for systolic BP and less precise: 1.1 (95% CI: 0.0, 2.2) mmHg and 0.8 (95% CI: 0.2, 1.4) mmHg increases in systolic and diastolic BP, respectively. Exclusion of participants with high noise exposure (*L*_den_ > 65 dB, *n* = 506) marginally increased the estimates for PM_2.5_: 1.5 (95% CI: 0.6, 2.5) mmHg and 1.0 (95% CI: 0.4, 1.5) mmHg for systolic and diastolic BP, correspondingly. Results for PM exposures modeled in quartiles were consistent with monotonic relationships (Figure 1).

**Figure 2:**
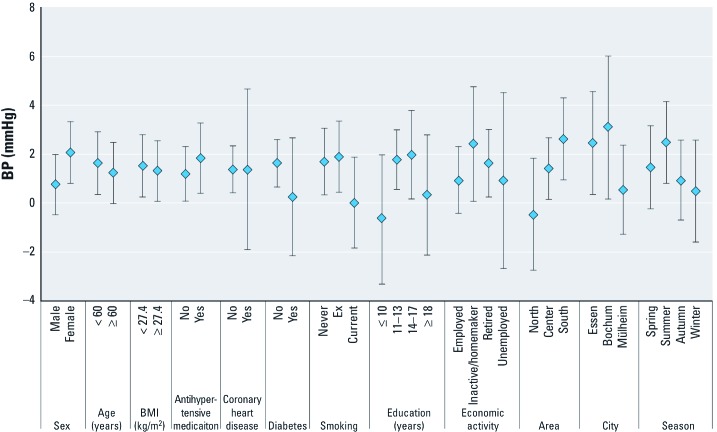
Effect of yearly mean PM_2.5_ IQR (2.4 μg/m^3^) on systolic BP (mmHg) (with 95% CI) in subgroups, using interaction terms (*n* = 4,291); adjusted for short-term PM, temperature, season, time trend, traffic proximity, sex, age, BMI, alcohol consumption, smoking, passive smoking, and physical activity.

Individual risk factors did not clearly modify the effect of PM_2.5_ on BP, although point estimates were increased somewhat for female sex, nondiabetics, nonsmokers, and participants with 11–17 years of education [representing medium SES in this cohort; for systolic BP, see Figure 2; for numeric values and interaction *p*-values for systolic and diastolic BP, see Supplemental Material, Table 4 (http://dx.doi.org/10.1289/ehp.1103564)]. Higher effects were also seen in those participants who were likely to spend more time at home, such as homemakers and retired subjects, but not for unemployed. Effect estimates were somewhat higher in the southern region, in the cities of Essen and Bochum, and in the spring and summer period.

Residential proximity to high road traffic was linked to elevated systolic and diastolic BP with higher point estimates and linear trend in subjects living close to high heavy-duty traffic, but estimates were imprecise because of small numbers in the exposure groups or because of loss of information using a categorized variable [see Supplemental Material, Tables 5 and 6 (http://dx.doi.org/10.1289/ehp.1103564)]. Long-term road traffic noise exposure > 60 dB at the residence was linked to higher BP, but estimates were again imprecise and generally lower than for traffic proximity and long-term PM exposure (see Supplemental Material, Table 7).

**Figure 3:**
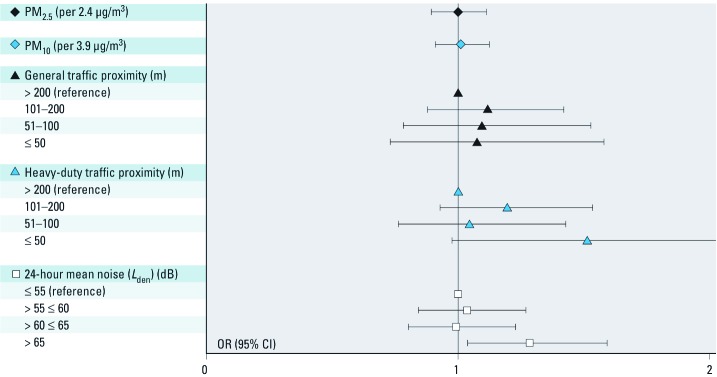
OR for hypertension (with 95% CI) by IQR increase PM_2.5_ and PM_10_ (µg/m^3^), by proximity to general-duty and heavy-duty traffic and by levels of road traffic noise (*n* = 4,291); adjusted for short-term PM, traffic proximity (or road traffic noise in the corresponding model), temperature, season, time trend, sex, age, BMI, alcohol consumption, smoking, passive smoking, and physical activity.

We found no association of 1-year PM with prevalence of hypertension (Figure 3).

Residential proximity ≤ 50 m to high heavy-duty traffic and long-term road traffic noise > 65 dB were associated with a somewhat higher prevalence of hypertension: odds ratios (ORs) = 1.51 (95% CI: 0.98, 2.34) and 1.28 (95% CI: 1.04, 1.59), respectively.

## Discussion

Our study shows a positive association of long-term exposure to urban background PM air pollution with systolic and diastolic BP. The observed relationship was independent of long-term exposure to road traffic noise and robust to the inclusion of a large number of potential confounders, including daily changes of PM, O_3_, and temperature. Traffic-related exposures (residential proximity to high traffic and traffic noise) trended toward higher BP and an elevated prevalence of hypertension.

Possible pathways through which PM could elevate BP include the elicitation of oxidative stress, systemic proinflammatory responses, and the activation of pulmonary reflexes leading to increased sympathetic tone, potentially causing arterial remodeling (Brook et al. 2009; Heagerty et al. 2010; Mills et al. 2009; Mulvany 2002). Autonomic nervous system imbalance after activation of pulmonary reflexes is most probably the responsible mechanism for acute effects of PM on BP, whereas systemic inflammation and vascular dysfunction involve a longer time of onset (Brook et al. 2009). A rise in intravascular pressure causes increased wall stress on the arterial wall, which then stimulates the activation of the medial smooth muscles in the arterioles. If this happens repeatedly, hypertrophic remodeling of the resistance vessels results in an increase in the medial thickness, which then leads to an increased total peripheral resistance and a fixation of elevated BP. Similarly, an increase in blood flow may lead to vasodilatation, which increases wall stress, leading to hypertrophic remodeling as well (Heagerty et al. 2010; Mulvany 2002). In eutrophic remodeling, neurohumoral activity as affected by PM activation of, for example, pulmonary reflexes leads to vasoconstriction and increased BP, but wall stress remains normal by decreasing the diameter of the vessel and increasing the wall thickness (Heagerty et al. 2010; Mulvany 2002).

The estimated effects in our study population are smaller than those found in a recent study from Asia (Chuang et al. 2011), where per IQR change in PM_2.5_ of 20.4 μg/m^3^, which is almost 10 times higher than in our study, systolic BP increased by 32.08 mmHg and diastolic BP increased by 31.29 mmHg. The higher effect size found by Chuang et al. (2011) might be explained by differences in age, ethnicity, and lifestyle of the study populations, differences in exposure assessment, and so on. Auchincloss et al. (2008) estimated a 1.0-mmHg increase in systolic BP with a 10-μg/m^3^ increase in mean monthly PM_2.5_ in a North American population. This estimated effect is smaller than ours; however, considering that yearly mean is less variable than monthly mean, which is reflected in a smaller IQR and exposure contrast in our study, the observed effects are quite similar.

Although we estimated a small absolute change in BP in our study, it is of potential public health importance. High BP is the most important determinant of cardiovascular mortality, and even small changes on the population level can lead to a high burden of attributable disease (Chobanian et al. 2003; Ezzati et al. 2002). An exposure contrast of only a few micrograms per cubic meter of PM_2.5_ in the urban background concentration may therefore lead to a shift in the population distribution of BP similar in effect size to the dietary salt reductions (Hooper et al. 2004). From this perspective, levels of urban background air pollution can be seen as a target for population-based preventive strategies (Rose 1985). Furthermore, we did not find evidence of a departure from a linear relationship between PM and BP, which supports the importance of reducing PM exposures at all levels. High BP also plays a major role in the development and progression of atherosclerosis. Our findings point to PM-related BP increases as one possible, although not exclusive, mechanism by which PM air pollution may lead to atherosclerosis, as suggested in recent studies (Bauer et al. 2010; Hoffmann et al. 2007; Künzli et al. 2010).

No single highly susceptible group could be identified in our analysis of subject characteristics. Rather, the estimated effect seemed consistent across the population. We found no clear effect modification of the PM effect by season, but the estimate was somewhat higher in spring and summer. This observation is similar to results of Choi et al. (2007), who reported PM effects on BP only during the warm season. Possible explanations include *a*) a different chemical composition of the PM mixture during the warm season, when, in the absence of household heating, traffic emissions cause a greater fraction of the overall particle mass or *b*) varying ventilation patterns in warm and cold seasons, resulting in different personal exposure. We estimated higher within-city effects for Essen and Bochum compared with Mülheim, similar to our previous analysis of fine PM and carotid intima-media thickness (Bauer et al. 2010). One possible explanation is the nature of the PM mixture. Exposure in Mülheim is characterized by the more even distribution of regionally transported PM, whereas in Bochum and Essen, local heavy industry contributes to within-city variations of primary industrial emissions (Bauer et al. 2010).

The spatial distribution of traffic-related PM is on a small scale for up to 400 m from a road (Zhou and Levy 2007). The EURAD model estimates an average background exposure in 1-km^2^ grid cells. In order to capture the small-scale traffic-related exposures, we therefore included traffic indicators as surrogates for the combined exposure to freshly produced traffic-related PM and traffic noise. Alternatively, we included modeled residential traffic noise exposure, making use of the mandatory European Union noise maps. Although adjustment for proximity to high traffic or traffic noise did not influence main effect estimates for urban background PM on BP, our results suggest a possible association of residential proximity to high traffic and high traffic noise with BP and prevalence of hypertension. We consider these findings to be indicative of traffic-related effects on a small spatial scale, independent of and in addition to the effects of urban background air pollution. In future analyses, we plan to investigate association between the local exposure to traffic-related air pollution and arterial BP more precisely, using different techniques for exposure assessment.

Our results on road traffic noise exposure are in line with the recent evidence (Bluhm et al. 2007; Bodin et al. 2009). Because of relatively small numbers of highly exposed participants, effects of noise exposure on BP were not estimated precisely enough in our study to reach definitive conclusions. An assessment of personal characteristics (e.g., impaired hearing) and of individual measures to reduce traffic noise exposure (type of windows, location of bedroom, ventilation habits) will help to clarify the role of chronic noise exposure on BP in future analyses. The positive associations of traffic indicators with BP and prevalence of hypertension, independent of the effect of background PM, may also indicate an effect of traffic-related PM.

The lack of a clear association of background PM exposure with prevalent hypertension might be due to loss of information when using a dichotomized outcome instead of a continuous outcome like BP. Another possible explanation includes the misclassification of the outcome hypertension, which was defined as a combination of measured BP and intake of drugs used for antihypertensive treatment. Some of these drugs have overlapping indications (e.g., diuretics or angiotensin-converting enzyme inhibitors might have been prescribed for heart failure instead of hypertension), leading to a false-positive assignment of hypertension in some cases. Additionally, local traffic-related PM, which is not captured in our PM model, may have stronger health effects than the mixture of background air pollution. This is supported by several studies that have shown an association of markers of subclinical atherosclerosis or cardiac function with traffic proximity but not with PM (Adar et al. 2010; Hoffmann et al. 2007; Künzli et al. 2010; Van Hee et al. 2009).

One limitation of our study is its cross-sectional design, which precludes a causal interpretation of the complex relationship between different risk factors for hypertension, BP, and antihypertensive medication. If PM actually caused an increase in BP, one would expect a higher use of antihypertensive medication in highly exposed subjects, assuming the same level of medical care. The intake of antihypertensive medication, however, decreases BP. If only this mechanism were in place, one would expect a negative or null association of PM and BP in the medicated participants. However, because the hypothesized effect of long-term PM exposure on BP is expected to be small relative to the effect of individual characteristics (e.g., age, BMI, physical activity), we assumed that its impact on the presence or absence of antihypertensive medication is likewise small compared with other risk factors. In sensitivity analyses, we explored the association of long-term PM exposure and BP with different model specifications, such as including and excluding antihypertensive medication from the adjustment set. Also, we limited the analysis to participants who did not use antihypertensive medication, thereby selecting possibly less susceptible individuals. None of these measures had an effect on the main estimate, but further analyses using follow-up data with repeated measurements of BP and incident hypertension will shed more light on this problem.

We did not explore the association of specific components of PM (e.g., black smoke) with BP, thus we are not able to identify the pathogenic constituents of urban background PM. Because effects of traffic exposure were observed mostly in relation to heavy-duty traffic consisting of diesel-powered engines, exploration of the compounds of air pollution and investigation of the effects of smaller particles should be the direction of future research. Other modeling techniques, such as land use regression models, which can model traffic-related air pollution on very small spatial scales of 10–20 m, are complementary to the chemistry transport model used here, which models the average exposure in a 1-km^2^ grid cell.

## Conclusions

We found an association of long-term exposure to PM with increased arterial BP in a population-based sample. This finding supports our hypothesis that long-term PM exposure may promote atherosclerosis with air-pollution–induced increases in BP being one possible biological pathway.

## Supplemental Material

(143 KB) PDF
