# Supplementary material for: Long-Term Urban Particulate Air Pollution, Traffic Noise, and Arterial Blood Pressure
Source: Environ Health Perspect. 2011 Aug 9;119(12):1706–11. doi: 10.1289/ehp.1103564 (PMC3261981; doi:10.1289/ehp.1103564)
Supplement: (143 KB) PDF [file ehp.1103564.s001.pdf]

Supplemental material

Title: Long-Term Urban Particulate Air Pollution, Traffic Noise and Arterial Blood Pressure

Authors: Kateryna Fuks, Susanne Moebus, Sabine Hertel, Anja Viehmann, Michael Nonnemacher, Nico Dragano, Stefan Möhlenkamp, Hermann Jakobs, Christoph Kessler, Raimund Erbel, and Barbara Hoffmann, on behalf of the Heinz Nixdorf Recall Study Investigative Group

TABLE OF CONTENTS

ABBREVIATIONS 3

METHODS, ADDITIONAL DESCRIPTIONS 4

Environmental data 4

Outcome 4

Other covariates. 5

Model specifications 6

Statistical methods 8

REFERENCES 10

TABLES 12

Table 1 12

Table 2 15

Table 3 16

Table 4 17

Table 5 19

Table 6 20

Table 7 21

FIGURES 22

Figure 1 22

ABBREVIATIONS

BMI – body-mass index

BP – blood pressure

CI – confidence interval

IQR – interquartile range

MET – metabolic equivalent

PM – particulate matter

SES – socioeconomic status

METHODS, ADDITIONAL DESCRIPTIONS

Environmental data

Particulate matter. We used a spatio-temporal exposure model to assess individual short- and long-term air pollution exposure at the home address of the participants for each day of the baseline examination period (Ebel et al. 2007; Memmesheimer et al. 2002). Daily concentrations of particiculate matter (PM) with aerodynamic diameter <10µm (PM_{10}) and <2.5µm ($PM_{2.5}$), and ozone for the years 2000-2003 were modeled using the EURAD (European Air Pollution Dispersion) model for the whole study area (approximately 600 km^2). The EURAD model uses the official emission inventory data, including industrial sources, household heating, traffic and agriculture, hourly meteorology, and regional topography. Surface concentrations were calculated by dispersing emissions in horizontal strata, taking regional transport, chemical reactivity, mass transport between horizontal strata, and deposition into account. The primary model output estimated the average pollutant concentration in 1 km^2 grid cells. The primary output concentrations were then calibrated with measured PM_{10} concentrations from routine monitoring sites throughout the region (n=6), taking the nature of the monitoring station (traffic, industrial site, urban background, regional background) into account. Data from measurement stations were supplied by the North Rhine-Westphalia State Agency for Nature, Environment and Consumer Protection. We assigned the calibrated daily surface concentration of PM and ozone in the corresponding 1 km^2 grid cell to the participants' addresses, using a geographic information system (ArcView 9.2, ESRI, Redlands, CA, USA). We calculated mean of the 365 daily PM values prior to the participant's examination date to assess the individual long-term residential exposure.

Outcome

Blood pressure was recorded using an automated oscillometric device (Omron HEM-705CP; OMRON Corporation, Hoofddorp, the Netherlands), and a random-zero sphygmomanometer (Mark II;

Hawksley, Lancing, United Kingdom) (Stang et al. 2006). Measurement devices were regularly calibrated by the Bureau of Standards (Board of Weights and Measures). Study personnel were certified and regularly trained in measuring blood pressure according to the standards of the World Health Organization MONICA blood pressure recording protocol (Chobanian et al. 2003). Blood pressure was recorded three times with both types of measurement device for each participant. Due to the time constraints and personnel shortage, a reduced program of blood pressure recordings (two automated and one random-zero reading) was applied for about 13% of the study participants. Measurements were performed on the right arm, in a seated position with at least 5-minutes rest before and a 3-minute interval between the blood pressure readings. The cuff size was selected according to the upper arm circumference. Participants were randomly assigned to the order of measurement devices. Interval between the two devices was on average 22 minutes.

We calculated the mean of second and third measurements with automated oscillometric device as a main outcome (n=4134) (Stang et al. 2006). In case of missing values, we calculated mean of first and second or first and third readings (n=616), or used the single available reading (n=15). In the cases when all three measurements with automated oscillometric device were missing, the BP value was replaced with the mean of second and third measurements with a random-zero BP device (n=30) or, if also unavailable, with a BP value from the pre-stress phase of the ergometric stress testing.

Other covariates

We assessed current and previous smoking, and calculated the lifetime cumulative exposure in pack-years. Passive smoking combined exposure at home, at the workplace and in other places. Anthropometric measurements and calculations (height, weight, body-mass index (BMI)) were conducted according to standardized protocols. Amount of alcohol intake were assessed by questionnaire and given as number of drinks per week (0.25 l beer, 0.1 l wine, or 0.02 l spirits).

Participant's physical activity was assessed by questionnaire and expressed as weekly energy expenditure in metabolic equivalents (MET). We defined coronary heart disease as a self-reported history of a myocardial infarction or coronary intervention. Diabetes mellitus was defined as prior physician diagnosis of diabetes or use of antidiabetic drugs or random blood glucose ≥11.1 mmol/L (≥200 mg/dL) or fasting blood glucose ≥7 mmol/L (≥126 mg/dL). We assessed individual-level socioeconomic status (SES) as years of formal education, categorized into four categories with the highest of ≥18 years (equivalent to a university degree) and the lowest category of ≤10 years (equivalent to a basic school degree and no vocational training) (United Nations Educational, Scientific, and Cultural Organization 1997), and economic activity (employed, retired, unemployed, or economically inactive).

Model specifications

We identified possible confounders using causal diagrams (Glymour and Greenland 2008). We specified the most likely temporal relations between variables based on prior biological and epidemiological knowledge and derived adjustment sets (see Figure 1). The following model specifications were used:

1. Crude model: We estimated the effect in the crude model, including only main exposure (1-year PM) and outcome, to investigate the scope and direction of the unadjusted effect and to be able to observe the effect of inclusion of potential confounding variables.
2. The basic model included the main exposure (1-year PM), short-term environmental variables (spatially resolved 3-day lag of PM, 3-day mean temperature, season, time trend), traffic proximity, and neighbourhood-level SES (city, area).
3. Main model: because the basic model is based on many assumptions regarding the relationship between area-level variables and the individual-level covariates, and because the adjustment for

area-level covariates is crude and might lead to relevant residual confounding, we included the main exposure, short-term environmental variables, traffic proximity, sex, age, and individual-level life-style covariates (BMI, alcohol consumption, smoking, passive smoking, physical activity).

As alternative model specifications, we used traffic noise and individual SES instead of traffic proximity in models 2 und 3.

4. Extended models:

4.1. Anti-hypertensive medication is a result of increased blood pressure, which itself is a result of the presence or level of various risk factors, including environmental and life-style characteristics. The complex temporal relationship between different causes of hypertension, blood pressure and antihypertensive medication cannot be addressed in a cross-sectional study design alone. However, since the hypothesized effect of long-term PM exposure on blood pressure is expected to be small relative to the effect of individual characteristics (i.e. age, BMI, physical activity, etc), we assumed that its impact on the presence of absence of antihypertensive medication is likewise small compared with other risk factors. As a sensitivity analysis in an extended model, we included antihypertensive medication in a separate step.

4.2. According to recent evidence, diabetes mellitus might also be an intermediate on the biologic pathway from air pollution exposure to high blood pressure (Krämer et al. 2010). Therefore, we included diabetes in a separate step.

4.3. It has been shown recently, that ozone might have an acute negative effect on blood pressure (Hoffmann et al. 2010). We therefore added short-term ozone exposure to the main model in a separate step.

4.4. To study the between- and within-area contrasts more explicitly, we conducted an extended analysis with a model that included both area-level and individual-level confounders, thereby reflecting the within-city effect of the basic model in a fully adjusted model.

Statistical methods

We included participants with non-missing information on exposure, outcome, and covariates (n=4,291). Correlations between PM exposures, traffic proximity, road traffic noise, short-term environmental variables were assessed with Spearman's rank correlation coefficient. Traffic proximity was entered as a continuous variable, and in the sensitivity analysis it was also truncated at 400 m, which did not change correlation coefficients. Long-term road traffic noise was entered in original 5-dB categories. We analyzed the association of PM with BP and hypertension using generalized additive models and logistic models, respectively. Systolic and diastolic BP were modeled continuously, hypertension as dichotomous outcome. $PM_{2.5}$ and PM_{10} were included as linear terms, because prior evidence suggests a linear exposure-response relationship between PM and cardiovascular outcomes. The effect of PM on BP and hypertension was estimated per inter-quartile range (IQR) increase of the exposure metrics. Continuous covariates which did not display departure from a linear relationship with the outcome were entered as linear terms, others were handled as splines. When more than one variable was available to control for possible confounding (for example, BMI and waist circumference as highly correlated anthropometric measurements), we made decisions about inclusion based on the model fit (partial F-test) (Kleinbaum et al. 2008).

Among the short-term environmental variables, the best-fitting short-term lag times and averages were found using the "empty" model without exposure and other covariates. The 3-day lag was used for $PM_{2.5}$ and PM_{10}, the 3-day moving average for temperature, and the 4-day moving average for ozone. The long-term background concentration of PM in the respective grid cell was subtracted from the short-term values before inclusion into the model to include only the acute deviations from the long-term average concentration.

Time trend, BMI, pack-years of smoking, and age (latter two only for diastolic BP as outcome) were entered as penalized cubic splines (Hastie and Tibishirani 2007). We assumed four degrees of freedom for the time trend, and three degrees of freedom for other non-linear terms.

REFERENCES

Chobanian AV, Bakris GL, Black HR, Cushman WC, Green LA, Izzo JL Jr, et al. 2003. Seventh report of the Joint National Committee on Prevention, Detection, Evaluation, and Treatment of High Blood Pressure. Hypertension 42(6):1206–1252.

Ebel A, Memmesheimer M, Jakobs HJ, Feldmann H. Advanced air pollution models and their application to risk and impact assessment. 2007. In: Air, water and soil quality modelling for risk and impact assessment (Ebel A., Davitashvili T., ed). Netherlands: Springer, 83–92.

Glymour MM, Greenland S. 2008. Causal diagrams. In: Modern epidemiology. (Rothman KJ, Greenland S, Lash TL, ed). 3rd ed. Philadelphia: Lippincott–Raven, 183–209.

Hastie TJ, Tibshirani RJ. 2007. Generalized additive models. London: Chapman and Hall.

Hoffmann B, Luttmann-Gibson H, Cohen A, Suh H, Coull BA, Schwartz J, Stone PH, Horton W, Gold DR. 2010. Higher temperatures and ozone reduce blood pressure but not arterial stiffness in diabetic patients [abstract]. Am J Respir Crit Care Med 181:A1730.

Kleinbaum DG, Kupper LK, Nizam A, Muller KE. 2008. Applied Linear Regression and Other Multivariable Methods, 4th ed. Belmont: Thomson.

Krämer U, Herder C, Sugiri D, Strassburger K, Schikowski T, Ranft U, et al. 2010. Traffic-related air pollution and incident type 2 diabetes: results from the SALIA cohort study. Environ Health Perspect 118(9):1273-1279.

Memmesheimer M, Friese E, Ebel A, Jakobs HJ, Feldmann H, Kessler C, Piekorz G. 2004. Long–term simulations of particulate matter in Europe on different scales using sequential nesting of a regional model. Int J Environ Pollut 22:108–102.

North Rhine–Westphalia State Agency for Nature, Environment and Consumer Protection. 2011. Available: http://www.lanuv.nrw.de/ [accessed 10 February 2011].

Stang A, Moebus S, Möhlenkamp S, Dragano N, Schmermund A, Beck E–M, et al. 2006. Algorithms for converting random–zero to automated oscillometric blood pressure values, and vice versa. Am J Epidemiol 164(1):85–94.

United Nations Educational, Scientific, and Cultural Organization. 1997. International Standard Classification of Education. Paris: United Nations Educational, Scientific, and Cultural Organization.

TABLES

Supplemental material, Table 1: Baseline characteristics (2000-2003) of the analyzed (n=4,291) and excluded (n=523) participants of the Heinz Nixdorf Recall Study.

Variable [metric]	Analyzed (n=4291) Mean ± SD (IQR) N (%)	Excluded (n=523) Mean ± SD (IQR) N (%)
**Long-term exposure**		
Mean $PM_{2.5}$ [µg/m^3]	16.7 ± 1.6 (2.4)	16.0 ± 1.5 (2.1)
missing[a]	-	5
Mean PM_{10} [µg/m^3]	20.7 ± 2.6 (3.9)	20.2 ± 2.7 (4.5)
missing	-	5
Traffic proximity, general [m]		
≤50	131 (3.1)	14 (2.7)
>50-100	178 (4.1)	20 (3.9)
>100-200	365 (8.5)	32 (6.2)
>200	3617 (84.3)	457 (87.2)
missing	-	9
Traffic proximity, heavy-duty vehicles [m]		
≤50	107 (2.5)	15 (2.9)
>50-100	199 (4.6)	28 (5.4)
>100-200	334 (7.8)	34 (6.6)
>200	3651 (85.1)	446 (85.0)
missing	-	9
24-hour mean noise (Lden) [dB(A)]		
≤55	2757 (64.3)	338 (64.6)
>55, ≤60	542 (12.6)	74 (14.1)
>60, ≤65	490 (11.4)	54 (10.3)
>65	502 (11.7)	57 (10.9)
**Short-term environmental variables**		
$PM_{2.5}$ (3-day lag) [µg/m^3]	16.5 ± 9.5	16.4 ± 9.5
missing	-	5
PM_{10} (3-day lag) [µg/m^3]	20.5 ± 11.3	20.4 ± 10.7
missing	-	5
Temperature (3-day mean) [^{o}C]	10.6 ± 7.4	7.3 ± 7.0
missing	-	5
O_3 (4-day mean) [µg/m^3]	37.4 ± 20.0	29.1 ± 18.0
missing	-	5
**Outcome**		
Systolic BP [mmHg]	132.7 ± 20.6	137.1 ± 22.2
missing	-	15
Diastolic BP [mmHg]	81.2 ± 10.8	83.4 ± 11.5
missing	-	14
Hypertension[b]	2444 (57.0)	125 (60.9)
missing	-	321
Antihypertensive medication	1550 (36.1)	88 (41.3)
missing		310

Supplemental material, Table 1 (cont.)

Variable [metric]	Analyzed (n=4291)<br>Mean ± SD (IQR)<br>N (%)	Excluded (n=523)<br>Mean ± SD (IQR)<br>N (%)
	Covariates	
Men	2147 (50.0)	248 (47.4)
Age [years]	59.7 ±7.8	59.5 ± 8.1
Coronary heart disease	290 (6.8)	37 (7.1)
Diabetes mellitus	594 (13.8)	62 (11.9)
BMI [kg/m^2]	27.9 ± 4.6	27.8 ± 4.8
missing[a]	-	29
Weekly physical activity [10^3 MET]	1.3 ± 2.1	1.1 ± 1.9
missing	-	87
Weekly alcohol intake [drinks][c]		
0	2801 (65.3)	207 (51.8)
1-3	2131 (49.7)	75 (18.8)
>3	749 (17.5)	118 (29.5)
missing	-	123
Passive smoking	1533 (35.7)	208 (52.0)
missing	-	21
Smoking status		
Never-smoker	1789 (41.7)	225 (43.9)
Ex-smoker	1513 (35.3)	149 (29.0)
Current smoker	989 (23.1)	139 (27.1)
missing	-	10
Education		
≤10 years	461 (10.7)	86 (17.0)
11-13 years	2390 (55.7)	286 (56.4)
14-17 years	970 (22.6)	98 (19.3)
≥18 years	470 (11.0)	37 (7.3)
missing	-	16
Economic activity		
Employed	1716 (40.0)	212 (41.7)
Inactive, homemaker	590 (13.8)	73 (14.4)
Retired	1719 (40.1)	184 (36.2)
Unemployed	266 (6.2)	39 (7.7)
missing	-	15

Supplemental material, Table 1 (cont.)

Variable [metric]	Analyzed (n=4291)	Excluded(n=523)
	Mean ± SD<br>N (IQR)<br>(%)	Mean ± SD<br>N (IQR)<br>(%)
Area-level covariates		
City		
Bochum	1270 (29.6)	131 (25.3)
Essen	1421 (33.1)	233 (45.0)
Mülheim	1600 (37.3)	154 (29.7)
Area		
North	600 (14.0)	91 (17.6)
Center	2412 (56.2)	281 (54.2)
South	1279 (29.8)	146 (28.2)
missing		5

[a] The "missing" category is present only if there are missing values for the variable.

[b] Hypertension was defined as SBP≥140 mmHg or DBP≥90 mmHg, or antihypertensive medication.

[c] 0.25 l beer, 0.1 l wine or 0.02 l spirits.

Supplemental material, Table 2: Correlation between individual-level exposure to long- and short-term air pollution, proximity to high traffic and traffic noise among study population (n=4,291), baseline examination [Spearman's rank correlation coefficient].

Factor	Spearman's rank correlation coefficient							
	Long-term exposures				Short-term exposures			
	PM_{10}	Traffic proximity		L_{den}	$PM_{2.5}$	PM_{10}	t[a]	Ozone
		general	heavy-duty					
$PM_{2.5}$ (1-year mean) [µg/m^3]	0.84 ***	0.13***	0.10***	0.07***	0.15***	0.18***	0.03*	-0.01
PM_{10} (1-year mean) [µg/m^3]	1.0	0.24***	0.24***	0.13***	0.15***	0.26***	0.02	-0.02
Traffic proximity, general [m]		1.0	0.72***	0.31***	0.04*	0.08***	-0.01	-0.02
Traffic proximity, heavy-duty [m]			1.0	0.29***	0.02	0.08***	0.01	-0.01
Lden (24-hour mean noise)[b]				1.0	0.04**	0.06***	-0.02	-0.02
$PM_{2.5}$ (3-day lag) [µg/m^3]					1.0	0.94***	-0.20***	-0.28***
PM_{10} (3-day lag) [µg/m^3]						1.0	-0.22***	-0.29***
Temperature (3-day mean) [°C]							1.0	0.87***
Ozone (4-day mean) [µg/m^3]								1.0`

Significance level: *p<0.05, **p<0.01, ***p<0.001.

[a]Temperature, °C.

[b]Categories: 0-55, >55 and ≤60, >60 and ≤65, >65 and ≤70, >70 and ≤75, >75 dB.

Supplemental material, Table 3: Analysis of long-term $PM_{2.5}$ and systolic blood pressure. Results of the generalized additive model analysis showing the estimates of all covariates from the main model (n=4,291).

Factor	Estimate (95% CI)	
Intercept	71.3	(63.0 to 79.6)
$PM_{2.5}$, yearly mean [2.4 µg/m^3]	1.4	(0.5 to 2.3)
$PM_{2.5}$, 3-day lag[a] [µg/m^3]	0.0	(0.0 to 0.1)
Temperature, 3-day mean [^{o}C]	-0.2	(-0.3 to -0.1)
Season (spring[b])	0.9	(-0.4 to 2.1)
Traffic proximity, general [m]		
>200 (reference)	-	
<u><</u>50	1.1	(-2.2 to 4.3)
>50-100	0.4	(-2.4 to 3.3)
>100-200	1.1	(-1.0 to 3.1)
Sex		
Male (reference)	-	
Female	-8.4	(-9.7 to -7.1)
Age [years]	0.7	(0.6 to 0.8)
Smoking status		
Never- and ex-smokers (reference)	-	
Current smoker	-2.1	(-3.6 to -0.6)
Passive smoking	1.3	(-0.1 to 2.6)
Weekly alcohol intake [drinks]		
0 (reference)	-	
1-3	-0.8	(-2.4 to 0.8)
>3	2.0	(0.6 to 3.5)
Weekly physical activity [10^3 MET]	-0.2	(-0.4 to 0.1)
Time trend [per 100 days, linear]	0.0	(0.0 to 0.0)
Body-mass index [kg/m^2, linear]	0.6	(0.5 to 0.8)

[a]Long-term mean subtracted.

[b]Due to high intercorrelation of seasons and their correlation with temperature, we adjusted for spring vs. other seasons.

Supplemental material, Table 4: Effect of $PM_{2.5}$ [µg/m^3] (per IQR=2.4 µg/m^3) on arterial BP [mmHg] (with 95% CI) in subgroups, using interaction terms. Adjusted for short-term PM, temperature, season, time trend, traffic proximity, sex, age, BMI, alcohol consumption, smoking, passive smoking, and physical activity.

	Systolic BP		Diastolic BP	
Factor	Estimate(95% CI)	p-value[a]	Estimate(95% CI)	p-value
Sex				
male	0.8 (-0.5 to 2.0)	0.13	0.6 (-0.1 to 1.3)	0.24
female	2.1 (0.8 to 3.3)	-[b]	1.1 (0.5 to 1.8)	-
Age [yrs]				
<60	1.6 (0.3 to 2.9)	0.65	1.0 (0.3 to 1.7)	0.49
≥60	1.2 (0.0 to 2.5)	-	0.7 (0.0 to 1.4)	-
BMI [kg/m2]				
<27.4	1.5 (0.2 to 2.8)	0.80	0.8 (0.1 to 1.5)	0.80
≥27.4	1.3 (0.1 to 2.5)	-	0.9 (0.3 to 1.6)	-
Antihypertensive medication				
no	1.2 (0.1 to 2.3)	0.47	0.9 (0.3 to 1.5)	0.93
yes	1.8 (0.4 to 3.3)	-	0.8 (0.1 to 1.6)	-
CAD				
no	1.4 (0.4 to 2.3)	1.00	0.8 (0.3 to 1.4)	0.86
yes	1.4 (-1.9 to 4.6)	-	0.7 (-1.1 to 2.5)	-
Diabetes				
no	1.6 (0.7 to 2.6)	0.29	1.0 (0.4 to 1.5)	0.23
yes	0.3 (-2.2 to 2.7)	-	0.1 (-1.2 to 1.4)	-
Smoking				
never	1.7 (0.3 to 3.1)	-	1.0 (0.2 to 1.7)	-
ex	1.9 (0.4 to 3.3)	0.85	0.8 (0.0 to 1.6)	0.77
current	0.0 (-1.8 to 1.9)	0.14	0.8 (-0.3 to 1.8)	0.74

[a]p-value for interaction term.
[b]Reference category.

Table 4 (cont)

	Systolic BP		Diastolic BP	
Factor	Estimate(95% CI)	p-value[a]	Estimate(95% CI)	p-value
Education [yrs]				
≤10	-0.6 (-3.3 to 2.0)	0.60	-0.6 (-2.1 to 0.8)	0.22
11-13	1.8 (0.6 to 3.0)	0.30	1.0 (0.3 to 1.6)	0.33
14-17	2.0 (0.2 to 3.8)	0.29	1.4 (0.4 to 2.4)	0.63
≥18	0.3 (-2.1 to 2.8)	-	0.6 (-0.7 to 1.9)	-
Economic activity				
employed	0.9 (-0.4 to 2.3)	-	0.7 (-0.1 to 1.4)	-
inactive/homemaker	2.4 (0.1 to 4.8)	0.27	1.3 (0.0 to 2.6)	0.39
retired	1.6 (0.2 to 3.0)	0.47	1.0 (0.3 to 1.8)	0.48
unemployed	0.9 (-2.7 to 4.5)	0.99	0.1 (-1.8 to 2.1)	0.61
Area				
North	-0.5 (-2.8 1.8)	-	0.4 (-0.8 to 1.6)	-
Center	1.4 (0.2 to 2.7)	0.15	1.0 (0.3 to 1.7)	0.41
South	2.6 (0.9 to 4.3)	0.03	1.0 (0.1 to 1.9)	0.44
City				
Essen	2.5 (0.4 to 4.6)	-	0.7 (-0.4 to 1.8)	-
Bochum	3.1 (0.2 to 6.0)	0.71	1.5 (0.0 to 3.1)	0.36
Mülheim	0.5 (-1.3 to 2.4)	0.15	0.4 (-0.6 to 1.4)	0.68
Season				
spring	1.5 (-0.2 to 3.2)	0.48	1.3 (0.4 to 2.2)	0.06
summer	2.5 (0.8 to 4.2)	0.14	1.3 (0.4 to 2.2)	0.06
autumn	0.9 (-0.7 to 2.6)	0.74	0.7 (-0.2 to 1.5)	0.33
winter	0.5 (-1.6 to 2.6)	-	-0.1 (-1.2 to 1.1)	-

Supplemental material, Table 5: Estimated absolute increase in systolic blood pressure [mmHg] for categories of residential traffic proximity (n=4,291).

Model	Absolute increase in systolic blood pressure [mmHg]					
	Proximity to high general traffic [m]			Proximity to high heavy-duty traffic [m]		
	0-50	51-100	101-200	0-50	51-100	101-200
Crude	1.4 (-2.2 to 5.0)	0.9 (-2.2 to 4.0)	1.4 (-0.8 to 3.6)	3.9 (-0.1 to 7.8)	2.2 (-0.8 to 5.1)	1.7 (-0.6 to 4.0)
Basic[a]	0.8 (-2.7 to 4.4)	0.6 (-2.5 to 3.7)	1.2 (-1.0 to 3.5)	3.4 (-0.6 to 7.4)	1.9 (-1.1 to 4.9)	1.5 (-0.8 to 3.9)
Main[b]	1.1 (-2.2 to 4.3)	0.4 (-2.4 to 3.3)	1.1 (-1.0 to 3.1)	3.0 (-0.6 to 6.6)	1.4 (-1.3 to 4.1)	1.3 (-0.8 to 3.4)
Extended = Main						
+ medication	0.9 (-2.4 to 4.2)	0.3 (-2.6 to 3.1)	1.1 (-1.0 to 3.1)	2.6 (-1.0 to 6.2)	1.2 (-1.4 to 3.9)	1.2 (-0.9 to 3.3)
+ diabetes	1.0 (-2.2 to 4.3)	0.4 (-2.5 to 3.2)	1.2 (-0.8 to 3.2)	2.9 (-0.7 to 6.6)	1.2 (-1.4 to 3.9)	1.3 (-0.8 to 3.4)
+ O_3[c]	1.1 (-2.2 to 4.4)	0.4 (-2.4 to 3.2)	1.1 (-1.0 to 3.1)	3.0 (-0.6 to 6.6)	1.3 (-1.4 to 4.0)	1.2 (-0.9 to 3.3)
+ city, area	1.0 (-2.3 to 4.3)	0.4 (-2.4 to 3.3)	1.1 (-1.0 to 3.1)	3.0 (-0.7 to 6.6)	1.4 (-1.3 to 4.1)	1.3 (-0.8 to 3.4)

[a] Adjusted for long- and short-term PM, temperature, season, time trend, city and area of residence.

[b] Adjusted for long- and short-term PM, temperature, season, time trend, sex, age, BMI, alcohol consumption, smoking, passive smoking, and physical activity.

[c] Due to high correlation with ozone, short-term temperature was excluded from this adjustment set.

Supplemental material, Table 6: Estimated absolute increase in diastolic blood pressure [mmHg] for categories of residential traffic proximity (n=4,291).

Model	Absolute increase in diastolic blood pressure [mmHg]					
	Proximity to high general traffic [m]			Proximity to high heavy-duty traffic [m]		
	0-50	51-100	101-200	0-50	51-100	101-200
Crude	1.1 (-0.8 to 3.0)	-0.4 (-2.0 to 1.2)	0.8 (-0.4 to 1.9)	2.3 (0.2 to 4.4)	0.3 (-1.3 to 1.8)	0.4 (-0.8 to 1.6)
Basic[a]	0.9 (-0.9 to 2.8)	-0.5 (-2.1 to 1.1)	0.7 (-0.5 to 1.8)	2.2 (0.2 to 4.3)	0.2 (-1.3 to 1.8)	0.4 (-0.8 to 1.6)
Main[b]	0.9 (-0.9 to 2.7)	-0.8 (-2.3 to 0.8)	0.8 (-0.3 to 1.9)	1.9 (0.0 to 3.9)	-0.3 (-1.7 to 1.2)	0.5 (-0.6 to 1.7)
Extended = Main						
+ medication	0.9 (-0.9 to 2.6)	-0.8 (-2.3 to 0.7)	0.8 (-0.3 to 1.9)	1.9 (-0.1 to 3.9)	-0.3 (-1.7 to 1.2)	0.5 (-0.6 to 1.7)
+ diabetes	0.9 (-0.9 to 2.7)	-0.8 (-2.3 to 0.8)	0.8 (-0.3 to 1.9)	2.0 (0.0 to 3.9)	-0.2 (-1.7 to 1.2)	0.5 (-0.6 to 1.7)
+ O_3[c]	0.9 (-0.9 to 2.7)	-0.8 (-2.3 to 0.7)	0.8 (-0.3 to 1.9)	2.0 (0.0 to 3.9)	-0.2 (-1.7 to 1.2)	0.5 (-0.7 to 1.6)
+ city, area	0.9 (-0.9 to 2.7)	-0.8 (-2.3 to 0.8)	0.8 (-0.3 to 1.9)	2.0 (0.0 to 3.9)	-0.2 (-1.7 to 1.2)	0.5 (-0.6 to 1.7)

[a] Adjusted for long- and short-term PM, temperature, season, time trend, city and area of residence.

[b] Adjusted for long- and short-term PM, temperature, season, time trend, sex, age, BMI, alcohol consumption, smoking, passive smoking, and physical activity.

[c] Due to high correlation with ozone, short-term temperature was excluded from this adjustment set.

Supplemental material, Table 7: Estimated absolute increase in arterial blood pressure [mmHg] for categories of traffic noise exposure (Lden) (n=4,291).

	Absolute increase in arterial blood pressure [mmHg] per category of 24-hour mean noise (Lden) [dB]					
	systolic BP			diastolic BP		
	>55, ≤60	>60, ≤65	>65	>55, ≤60	>60, ≤65	>65
Crude	-1.0 (-2.9 to 0.9)	1.9 (-0.1 to 3.8)	0.9 (-1.1 to 2.8)	-0.5 (-1.5 to 0.5)	0.9 (-0.1 to 1.9)	0.9 (-0.1 to 1.9)
Basic[a]	-0.9 (-2.8 to 0.9)	1.6 (-0.4 to 3.5)	0.8 (-1.1 to 2.7)	-0.5 (-1.5 to 0.4)	0.8 (-0.2 to 1.8)	0.8 (-0.1 to 1.9)
Main[b]	-0.8 (-2.5 to 0.9)	1.3 (-0.5 to 3.1)	0.9 (-0.9 to 2.6)	-0.5 (-1.4 to 0.5)	0.8 (-0.2 to 1.8)	0.8 (-0.2 to 1.8)
Extended = Main						
+ medication	-0.8 (-2.6 to 0.9)	1.4 (-0.5 to 3.2)	0.8 (-1.0 to 2.6)	-0.5 (-1.4 to 0.5)	0.8 (-0.2 to 1.8)	0.7 (-0.2 to 1.7)
+ diabetes	-0.8 (-2.5 to 0.9)	1.3 (-0.5 to 3.1)	0.9 (-0.9 to 2.6)	-0.4 (-1.4 to 0.5)	0.8 (-0.2 to 1.8)	0.8 (-0.2 to 1.7)
+ O_3[c]	-0.8 (-2.5 to 1.0)	1.3 (-0.5 to 3.1)	0.9 (-0.9 to 2.7)	-0.5 (-1.4 to 0.4)	0.8 (-0.2 to 1.8)	0.7 (-0.2 to 1.7)
+ city, area	-0.8 (-2.5 to 1.0)	1.5 (-0.3 to 3.3)	0.9 (-0.9 to 2.7)	-0.4 (-1.4 to 0.5)	0.8 (-0.2 to 1.8)	0.8 (-0.2 to 1.8)

[a] Adjusted for long- and short-term PM, temperature, season, time trend, city and area of residence, education, and economic activity.

[b] Adjusted for long- and short-term PM, temperature, season, time trend, sex, age, education, economic activity, BMI, alcohol consumption, smoking, passive smoking, and physical activity.

[c] Due to high correlation with ozone, short-term temperature was excluded from this adjustment set.

FIGURES

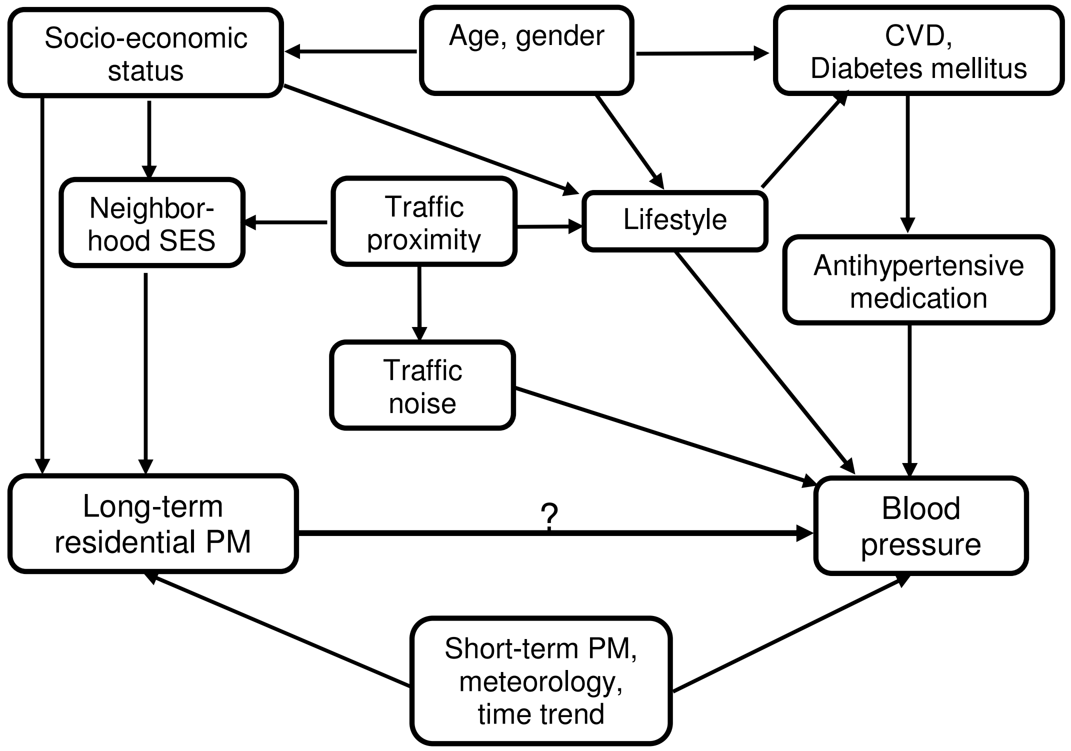

Supplemental material, Figure 1: Hypothesized association between exposure, outcome, and covariates in our study.
